# Ambient Cured Fly Ash Geopolymer Coatings for Concrete

**DOI:** 10.3390/ma12060923

**Published:** 2019-03-20

**Authors:** L. Biondi, M. Perry, C. Vlachakis, Z. Wu, A. Hamilton, J. McAlorum

**Affiliations:** 1 Department of Civil and Environmental Engineering, University of Strathclyde, Glasgow G1 1XJ, UK; m.perry@strath.ac.uk (M.P.); christos.vlachakis@strath.ac.uk (C.V.); andrea.hamilton@strath.ac.uk (A.H.); jack.mcalorum@strath.ac.uk (J.M.); 2 Department of Mechanical and Industrial Engineering, University of Toronto, Toronto, ON M5S 3G8, Canada; zitian.wu@mail.utoronto.ca

**Keywords:** concrete repair, geopolymers, alkali-activated materials, coating thickness, ambient curing, shrinkage, efflorescence, cracking, water transport

## Abstract

The reinforced concrete structures that support transport, energy and urban networks in developed countries are over half a century old, and are facing widespread deterioration. Geopolymers are an affordable class of materials that have promising applications in concrete structure coating, rehabilitation and sensing, due to their high chloride, sulphate, fire and freeze-thaw resistances and electrolytic conductivity. Work to date has, however, mainly focused on geopolymers that require curing at elevated temperatures, and this limits their ease of use in the field, particularly in cooler climates. Here, we outline a design process for fabricating ambient-cured fly ash geopolymer coatings for concrete substrates. Our technique is distinct from previous work as it requires no additional manufacturing steps or additives, both of which can bear significant costs. Our coatings were tested at varying humidities, and the impacts of mixing and application methods on coating integrity were compared using a combination of calorimetry, x-ray diffraction and image-processing techniques. This work could allow geopolymer coatings to become a more ubiquitous technique for updating ageing concrete infrastructure so that it can meet modern expectations of safety, and shifting requirements due to climate change.

## 1. Introduction

Coating technologies are an effective means of protecting concrete structures from chemical attack and rebar corrosion. Inorganic coatings in particular have been widely applied as anticorrosive and decorative materials for concrete and steel structures [1,2,3,4]. These materials show a high long-term durability even under acid and alkali attack and at elevated temperatures [5]. Emerging solutions for concrete protection based on alkali-activated materials, here referred to as geopolymers, show rapid setting and hardening, excellent bond strength and durability, low chloride permeability and high freeze-thaw and chloride resistances [5,6,7,8,9,10,11,12,13,14,15,16,17,18,19,20,21,22,23]. Geopolymers also possess an electrolytic conductivity, which can allow them to be simultaneously used as skin-sensors for structural health monitoring [24,25,26,27,28].

Regardless of the application, a good coating will be free of cracks and defects. Integrity is an ongoing issue regarding the development and practical application of geopolymer coatings. Some common concerns include cracking due to shrinkage, changes to setting times and efflorescence [8,29,30]. These issues are particularly acute when geopolymers are used in field conditions which are at the extremes of humidity or moisture scales.

In this work, we aimed to develop high-quality, ambient-temperature-cured fly ash geopolymer coatings. We wished to achieve this with minimal additional processing steps (such as fly ash grinding or the use of additives), so as to minimize manufacturing costs and complexity. This was challenging: geopolymers are typically cured at elevated temperatures to accelerate geopolymerisaton, as rapid curing allows coatings to achieve a higher early-age strength [12,31,32,33,34]. This can make geopolymers inconvenient to apply in the field, particularly in cooler climates. For this reason, researchers have been looking into ambient temperature curing of geopolymers [14,31,32,35,36,37]. In particular, Somna [31], Temuujin [14] and others [38,39,40] ground fly ash particles to improve their reactivity and promote room-temperature curing. Some researchers have studied the effects of calcium rich additives, such as slag, on curing in ambient conditions [36,41,42,43,44,45,46], while others have studied the effects of moisture [32]. These previous works have studied the ambient temperature curing of fly ash geopolymers cast in moulds. Their aim was to deliver geopolymers that serve a structural function. As such, they demonstrate high compressive strength and enhanced hardening; however, at the expense of reduced workability and increased drying shrinkage [14]. The work presented here is distinct, as we developed fly ash geopolymer coatings; thus, high workability and low shrinkage were key requirements, in addition to the performance requirements for non-structural repairs stated in the standard BS EN 1504-3:2005. Our primary objective in this work was to develop a non-structural repair material that can be later developed for use as a sensor, as in [24,25,26,27,28].

The notable contributions of this paper are four-fold: (i) we present an affordable process for ambient-temperature geopolymer synthesis that does not require additives or grinding; (ii) we outline the influence of prolonged mixing times on coating quality (previous studies use short mixing times under 10 min [31,32,37,47]); (iii) we study the influence of concrete substrate age on geopolymer coating quality (previous work has studied coatings on mature concretes only [8,48]), and (iv) perhaps most importantly, this paper provides a frank discussion of the challenges faced during geopolymer development—we hope that researchers, especially those new to the field, will find this discourse useful.

This paper begins with a description of geopolymer synthesis and the factors that influence coating integrity in Section 2. The materials and procedures used for geopolymer synthesis in this work, the testing methods and the analysis conducted are outlined in Section 3. Finally, results and further discussion are presented in Section 4 and Section 5.

## 2. Theory 

### 2.1. Geopolymer Fabrication

Geopolymers form as the result of several reactions between an alkaline activator and inorganic materials which are rich in silicon (Si), aluminum (Al) and oxygen (O). For an excellent introduction and description of geopolymer systems, readers are directed to [49]. Typical inorganic precursors for geopolymer synthesis include blast furnace slag, metakaolin and fly ash [33,49,50,51]. Geopolymers derived from fly ash, used in this work, offer a few key advantages over other geopolymer precursors, including:

- Higher workability, durability and strength: this is due to the lubricating and re-enforcing effect of unreacted fly ash particles [6,30].
- Low cost: as fly ash is a by-product of coal combustion, it is cheap and available in large volumes around the world [30]. While there is a looming shortage of fly ash for use as a concrete additive [52], the availability for geopolymer coatings (a low volume application) is still high, as one billion tons of fly ash are still produced annually worldwide in coal-fired steam power plants [53].

The alkaline activator solution (L) used in this work was rather typical: a combination of sodium hydroxide (SH) and sodium silicate (SS) [54]. The chemistry and resulting wet/cured properties of the fly ash powder (A) geopolymer were mainly defined by [33,55]:

1. The mass ratio, L/A;
2. The mass ratio, SH/SS;
3. The molarity of the SH (which typically ranges from 8–14 M).

While molarity can, to some extent, be selected based on safety considerations, the ratios L/A and SH/SS should be selected to match the chemical composition of the fly ash. This is a significant drawback for fly ash geopolymers, as: (i) fly ash composition can vary significantly between coal plant sources; and (ii) unlike with Portland cement systems, there are no simple numerical methods for geopolymer mix design, particularly for fly ashes [6,56]. The ratios used in this work, outlined in Section 3, were found through a process of trial and error over a testing matrix, and on the basis of literature findings, according to [47].

### 2.2. Factors Affecting Geopolymer Coatings on Concrete Substrates

Repair works, including coatings, aim to preserve or restore concrete structures [57]. The series of standards EN 1504 define the requirements for repair procedures and the properties of repair materials [58,59]. Geopolymers are physically compatible with concrete substrates in most of these regards [60]. However, there are technical issues that are particular to geopolymer coatings on concrete substrates, particularly those cured at ambient temperatures. Note that the issues outlined in the following subsections are common across most geopolymer systems, not just those made with fly ash precursors. 

#### 2.2.1. Shrinkage and Curing

For geopolymer coatings cured on concrete at ambient temperatures, shrinkage is the most significant issue [61]. Shrinkage is defined as a reduction in the volume of the geopolymer because of a loss of water: this is predominantly due to drying, but it can also occur when water is used up during geopolymerisation [62].

Drying shrinkage is the result of a loss of water from the geopolymer’s capillary pores. This loss of moisture causes the tension in the capillary pores to increase, resulting in a volume reduction in the specimen. When geopolymer coatings cure, they undergo this shrinkage while simultaneously binding to the underlying concrete substrate. The resulting confinement allows shrinkage strains to cause tensile and shear stresses. If significant shrinkage occurs before the coating has adequately cured, these stresses will exceed the strength of the geopolymer [63], leading to cracks and debonding, that undermine coating integrity.

**Figure 1:**
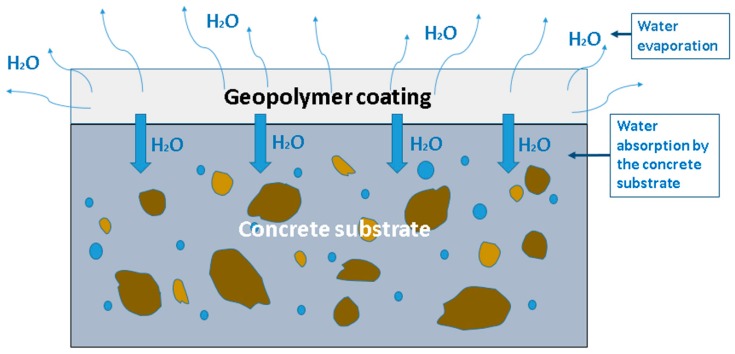
Illustration of the main water loss mechanisms from geopolymer coatings on concrete substrates.

For concrete coatings, there are at least two major contributors to drying shrinkage, illustrated in Figure 1. The first, affecting all geopolymers regardless of substrate, is that a low environmental humidity encourages water loss through evaporation. Indeed, it is well known that drying shrinkage can be reduced by curing geopolymers in hermetically sealed conditions [63,64]. However, the second mechanism for water loss is the diffusion of water from the moist geopolymer layer into the drier, porous, concrete substrate. Thus, control of the moisture content of the substrate is essential to control cracking [65]. 

As both drying mechanisms are surface-area dependent processes, the edges of geopolymer patches are particularly prone to cracking, as these edges can present an extra surface for evaporation. 

One clear way of tackling this issue is to accelerate the curing rate of the geopolymer coating (and this is why elevated temperatures are often used). Grinding fly ash particles to improve their reactivity and allow room temperature curing, as in [31], may appear to be another solution; however, this can in some cases lead to a higher shrinkage, due to the particle size being so fine that agglomerates form during mixing, resulting in a lower reaction rate which increases shrinkage [66]. While we are avoiding additives in this work, it is worth noting that plastic fibers can reduce shrinking and cracking, especially for sealed curing conditions [64].

#### 2.2.2. Adhesion, Workability and Setting Time

One of the most common ways to apply cementitious coatings to concrete in the field is by pumping and spray coating. There have also been lab studies that demonstrated (as is shown in this work) the application of geopolymer coatings with a trowel [7], and even some that applied coating using manufacturing methods such as three-dimensional (3D) printing [26]. Regardless of the method used, one must consider the interplay between a geopolymer’s adhesion to concrete, its workability and its setting time (and by extension, its behavior during curing and shrinkage). Geopolymers are highly tuneable materials, but the majority of mixes will display:

- High surface tension: this plays a key role in the ability of the materials to bind and stay bound to substrates. A reduction in surface tension may, in some cases, be required to ensure good adhesion [67]. With respect to geopolymer microstructure formation, the water to solid ratio can significantly affect the process of geopolymerization, and hence the properties of coatings, such as their workability and adhesion [5].
- Thixotropic Bingham plastic fluid behavior: most geopolymers show history-dependent rheological behavior, and can be kept in fluid form if subjected to constant shearing [13,68,69]. Their rheological behavior can also be tuned by altering the molar concentration of the sodium hydroxide and the ratio of silicate to hydroxide solutions [70].
- Setting times that are strongly dependent on chemical composition: the setting time of geopolymers can range from minutes to hours and depends on geopolymer composition. Setting times can be reduced by lowering Si/Al ratios, or by increasing calcium (Ca) content [61]. For systems with high Si/Al ratios, polymerisation is more likely to occur among silicate species; however, when Si/Al ratios are lowered, polymerisation is more likely to occur between aluminate and silicate species. As condensation among silicate species is slower than that between aluminate and silicate species, setting is delayed with higher Si/Al ratios [71].

The impact this has on coatings is that it can be challenging to independently tune adhesion, rheology, setting time and shrinkage behavior, as the properties of the system are highly interdependent. Therefore, previous work conducted at elevated temperatures may not always map onto efforts to produce geopolymer coatings that cure in ambient conditions.

#### 2.2.3. The Concrete Substrate

Previous studies have found that the adhesion of coatings more generally is strongly influenced by the roughness of the substrate surface, its water content and the mix composition of the coating material [72,73,74,75,76,77,78]. Rough surfaces on concrete substrates are preferred as greater bond performance is ensured [76,79]. Among the available surface preparation methods, a high bond strength can be achieved with sand-blasting and wire brushing [72]. Morgan [80] states that the degree of roughness and means of roughening both affect long-term performance. Before applying the geopolymer repair material Zanotti et al., for example, roughened the surface using the sandblasting technique [81]. 

Some authors [80,82,83] have shown the important role of the substrate during concrete-to-concrete repair work. A significant mismatch between substrate and repair concrete is a notable consideration if the repair is to resist the stresses induced by dimensional, mechanical and durability incompatibility. The surface of the substrate should have an open pore structure, to allow the absorption of the repair material into the substrate’s pore structure, thus enhancing the bonding mechanism. In geopolymer coatings, this is at odds with the requirements for reducing drying shrinkage, as an excessively dry substrate with open pores may absorb too much water from the coating [84].

Today, opinions diverge about the most appropriate practice when coating and repairing concrete substrates. Even between international codes of practice, recommendations are contradictory. The AASHTO-AGC-ARTBA Joint Committee recommends a dry surface for concrete, except on dry and hot summer days, while the Canadian Standards Association Standard A23.1 recommends wetting the surface for at least 24 h before casting the new concrete [72]. In some studies, saturated but surface dry conditions were considered to be the best solution [84]. 

#### 2.2.4. Efflorescence

A final issue, which can be particularly prevalent in ambient temperature cured geopolymers, is efflorescence. Efflorescence is the formation of white salt deposits, and it can unfortunately occur during attempts to manipulate geopolymer shrinkage, adhesion, workability and setting time. It has been found that efflorescence is due to many factors [30]: wet conditions, the reactivity of raw materials, the alkali metal type and reaction conditions. In particular, a high alkali content in the activator solution causes efflorescence in partially wet conditions [29,85]. Thus, geopolymer efflorescence is common at high humidity, and this is important because humidity cannot always be controlled in the field. Ambient temperature curing also makes efflorescence more likely, because the low temperatures reduce the dissolution rate of the fly ash by the alkaline solution. Therefore, any excess alkaline solution is more likely to induce crystallization on the surface [14]. 

## 3. Materials and Methods

### 3.1. Materials

**Figure 2:**
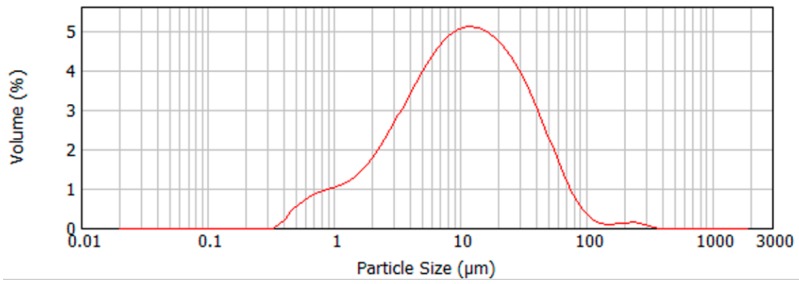
Particle size distribution of the fly ash.

In this work, geopolymers were synthesized from low calcium fly ash. According to standard BS EN 450, the fly used was class B for Loss On Ignition (LOI) of 2.0% to 7.0%, and category S for fineness (no more than 12% retained on the 45 micron sieve). Under US notation, according to ASTM C 618-19, the fly ash used in this work would be considered class F. The chemical compositions of the fly ash used is given in Table 1, along with the information on the source of the ash and its median particle size, measured using a Mastersizer 2000. Figure 2 shows the particle size distribution of the fly ash. The median value in Table 1 is the D50 or d (0, 5) value, defined as the intercept for 50% of the cumulative mass.

The alkaline activator used in this work was made by combining 10 wt% of 10 M sodium hydroxide solution (NaOH) and 24 wt% sodium silicate solution (Na_2_SiO_3_), with the NaOH/ Na_2_SiO_3_ ratio equal to 0.4. This is in accordance with our previous work [28] based on fly ash geopolymer coatings as skin sensors for concrete. The sodium silicate solution composition was made by 8.5 wt% Na_2_O and 27.8 wt% SiO_2_, in distilled water. The Na_2_O and SiO_2_ concentrations of the alkaline activator were 12.7 wt% and 19.9 wt%, respectively, and the remaining 67.4 wt% was deionized water. The activator was made 24 h prior mixing, to allow the heat of any exothermic reaction to dissipate. 

### 3.2. Methodology

#### 3.2.1. Geopolymer Synthesis 

The geopolymer binder was fabricated by combining the fly ash with the activator solution, with a Liquid/Solid ratio (L/S) = 0.5. According to Nedelikovic et al., L/S = 0.5 improves workability, without having a significant effect on compressive strength [86]. A higher quantity of liquid also produces a less viscous slurry, which can penetrate more easily into the surface of a dry concrete substrate [87].

**Figure 3:**
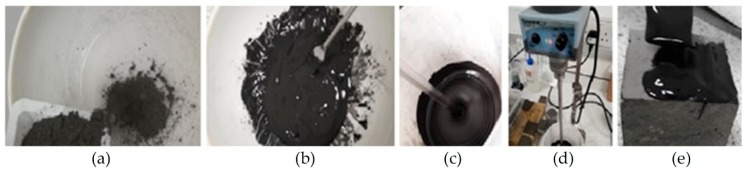
Geopolymer synthesis process: (**a**) adding fly ash powder into alkaline solution; (**b**) manually mixing the geopolymer binder by means of a spatula; (**c**,**d**) automatic mixing of the binder; (**e**) application of the binder onto concrete by means of a trowel.

Figure 3 summarizes the steps taken to mix and apply geopolymers. The mixing procedure consists of gradually adding the fly ash powder into a bowl containing the alkaline solution while continuously mixing (Figure 3a–c). Samples were either mixed manually (Figure 3b), or with an automatic mixer at 500 min^−1^ (Figure 3c,d). Geopolymer binders were mixed for between 10 min and 1 h before being applied to concrete substrates with a trowel (Figure 3e).

#### 3.2.2. Application to Substrate

Two thicknesses (here defined as *m*) of geopolymer coatings were applied to concrete substrates in this work. Thin coatings were around *m* = 1 mm, and thicker coatings were *m* = 3 mm. These thicknesses were chosen in accordance with the requirements for sensing [28] and the requirements for non-structural repairs outlined in standard BS EN 1504-3:2005. Structural repair coatings typically require much higher thicknesses (15 mm or 50 mm) [88].

To study any potential effects of the concrete substrate on coating integrity, we applied geopolymer coatings to concrete samples with varying age ranges:

- Newly cast, or “young” concrete samples, left to cure for 1–5 months;
- Intermediate-aged concrete samples, 5–12 months of curing;
- Old concrete samples, over 1 year of curing.

Our hypothesis was that the changing pore structure of the concrete substrate could affect moisture transport from the geopolymer layer, and thus coating integrity. As concrete matures, hydration progresses and capillary pore size and porosity decrease from the production of C-S-H. Bentz et al. commented that when the volume fraction porosity has been reduced to approximately 0.20, the pore space is no longer interconnected throughout the paste and that water transport is restricted; however, the small gel pores (<10 nm in diameter) remain filled at relative humidity (RH) values of 50% and higher [89,90]. As a greater percentage of filled pores results in less capillary suction, the more mature concrete might be expected to drain less water than the newer samples.

#### 3.2.3. Concrete Substrate Roughness

The surface roughness of each concrete substrate used was measured by 3D laser scanning (using a Micro Epsilon Scan Control 2700–100, an exposure time of 1 msec, 56 profiles per second, and 1600 buffered profiles). The values for surface roughness were determined by analyzing the root mean square deviation of the point cloud from a mean plane. Typical values of surface roughness for concrete samples are shown in Table 2. Values all correspond to the smooth surface that one would expect from untreated concrete [91].

#### 3.2.4. Curing Conditions for Geopolymers

Geopolymer specimens were batched and placed within one of two curing conditions, summarized in Table 3. Both patches were cured at 20 °C, and the relative humidity of batch 1 and batch 2 were 50% and 95%, respectively. Temperature and relative humidity (RH) were measured in lab conditions and shown to be relatively stable for batch 1; however, they were not tightly controlled. Meanwhile, batch 2 was cured in an environmental chamber in controlled conditions.

All geopolymer specimens in the batches were left to cure for 28 days. While geopolymers do tend to cure much faster than Portland cement mixes, we opted to use a prolonged curing duration in this work to ensure that patches were fully cured and stabilized in ambient conditions.

#### 3.2.5. Analysis Methods

Several tests were carried out on the fly ash powder and the geopolymer binder to characterize their properties, before mixing, during curing and after curing. These are summarized in the following sections.

##### 3.2.5.1. Vicat Needle Test

The setting time of geopolymer mixes was measured using the Vicat needle test, following the procedure outlined in BS EN 196, part 3 [92]. This test was conducted in order to define a suitable time to apply the geopolymer onto the concrete substrate, and to define the shelf life for our geopolymer mixes. While the Vicat needle test is a well-accepted and easy-to-use standard method used within ordinary Portland cement concrete mix design, it is less accurate than modern calorimetric and viscosity measurements and so results should be interpreted with caution.

##### 3.2.5.2. Isothermal Calorimetry

A thermal analysis, together with an evaluation of setting time using the Vicat needle test, can be used to define an optimized time for applying geopolymer coatings to concrete substrates. In this work, an isothermal calorimeter (Calmetrix, I-CAL 4000 HPC) was used to measure the temporal dependence of the heat produced by the exothermic reactions occurring in the geopolymer from immediately after mixing up to 3 days. Tests were conducted three times for each geopolymer mix tested, with the averaged heat curve presented in the results.

##### 3.2.5.3. X-Ray Diffraction Analysis

X-ray diffraction (XRD) analysis was carried out on samples of fly ash, on geopolymer layers and on geopolymer coatings, which had demonstrated efflorescence. All XRD data was collected using a Bruker D8 Advance instrument. Data for Rietveld refinement was collected in Bragg-Brentano geometry from 12°–75° 2theta with an increment of 0.02 °/s and a step time of 0.8 s. using a divergence slit of 0.23°. A Ni β filter was placed in the incident beam path. The sample was rotating at 30 rpm and a knife edge collimator was used to reduce air scattering. 

The efflorescence was analyzed intact on a sample of geopolymer. The efflorescence could not be easily removed for conventional powder analyses and was analyzed in situ in the XRD using a Goebel mirror. Data was collected from 10°– 60° 2theta with an increment of 0.02 °/s and a step time of 8 s. Quantitative analysis was carried out using the internal standard method by adding 10 wt% silicon to the ground powder samples, and TOPAS software. ICSD structure files used in the refinement are listed in caption of Figure 15. The method proposed by Williams and van Riessen [93], which uses the intensity ratio of the 210 and 120 reflections (I_210_/I_120_) of mullite to estimate the mineral’s Si/Al ratio, was used to choose the most appropriate structure file to include (ICSD collection code 66449) in the TOPAS refinement. They showed a linear relationship between x in the mullite general formula (Al_4+2x_Si_2-2x_O_10-x_) and the 210/120 reflection intensity which was used here to calculate x = 0.28 for the Cemex fly ash. 

##### 3.2.5.4. Compressive Strength

Compressive strength tests were conducted with a small loading cell with the speed of 2 mm/min on geopolymer cubes of side 30 mm. Tests were conducted after 1 day, 2 days, 3 days, 4 days, 7 days, 14 days and 28 days. The intention here was to demonstrate the evolution of strength, rather than strictly comply with strength-testing standards.

##### 3.2.5.5. Visual Inspection and Quantification of Cracks

**Figure 4:**
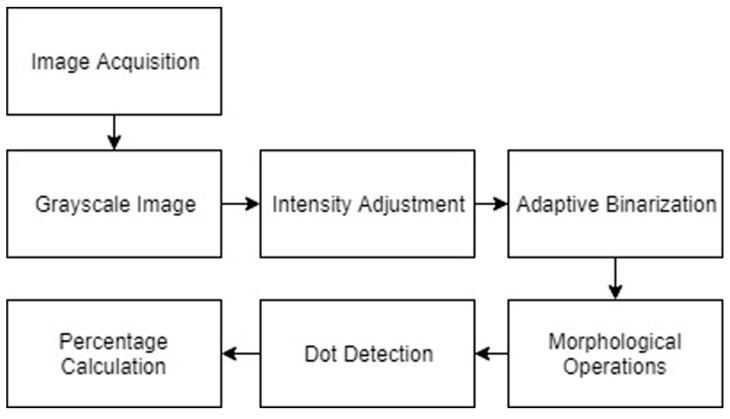
Steps of the image processing method.

A visual inspection was often enough to provide a binary “yes/no” assessment of whether a geopolymer coating had cracked after curing. However, to quantify the relative levels of cracking between specimens in a less subjective manner, we developed a simple image processing technique outlined in Figure 4.

The original sample images were taken using a DSLR (Digital Single-Lens Reflex) camera with a set distance between the sample and the focal lens. The image was then cropped so that all samples produce images of the same size for ease of comparison. The color images obtained were converted to grayscale images. This eliminated colors during further processing, while preserving the intensity of each pixel in the image with a grayscale level. 

**Figure 5:**
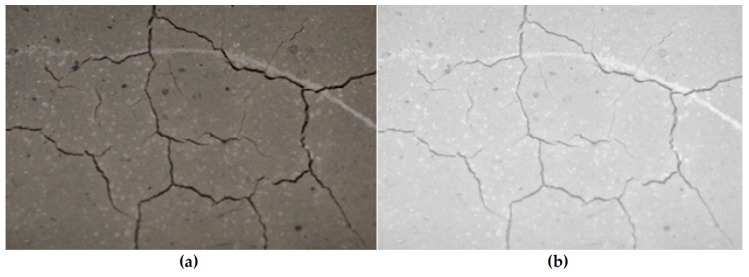
(**a**) Original image and (**b**) grayscale image after intensity adjustment.

In order to ensure that the cracks are the darkest part of the image, a pre-processing step of intensity adjustment was required. The bottom 1% and the top 1% of all pixel values were saturated to increase the contrast of the output image. By identifying and intensifying the pixels below the mean grayscale value in the image, a clearly distinguished foreground of cracks was obtained, as shown in Figure 5.

During the image acquisition step, the inconsistent amount of light in the background was difficult to avoid. In order to correct for this non-uniform illumination, adaptive binarization was used. By applying Bradley’s method, each pixel in the integral image was compared to the average grayscale level of its surrounding pixels and set to a binary value accordingly [94].

**Figure 6:**
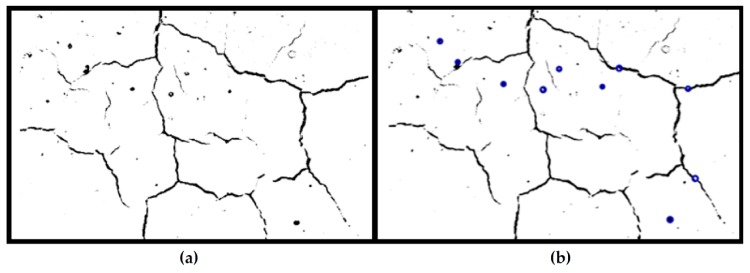
Binary images: (**a**) morphological operation; (**b**) dot detection.

Due to the special characteristics of cracks in our samples, morphological operations and dot detection could be used to reduce noise that will interfere with the final quantification of cracking [95]. One pixel with more than four connected neighborhoods is seen as one element. By removing elements that contained fewer than 10 pixels, more noise could be eliminated from the image. Dot detection was used, since most of our samples had dark bubbles that were difficult to distinguish from real cracks in the previous step. By utilizing the circular Hough transform, the round objects could be identified and eliminated from the calculation. The Hough method is one of the standard methods for image recognition [96]. The images after morphological operations and dot detection are shown in Figure 6.

The final step was to calculate the percentage of dark pixels (cracks) over the area of the whole image, yielding a quantified and less subjective result for the levels of cracking in each sample. This result was provided as a percentage of the image that showed cracks.

## 4. Results and Discussion

### 4.1. Compressive Strength

**Figure 7:**
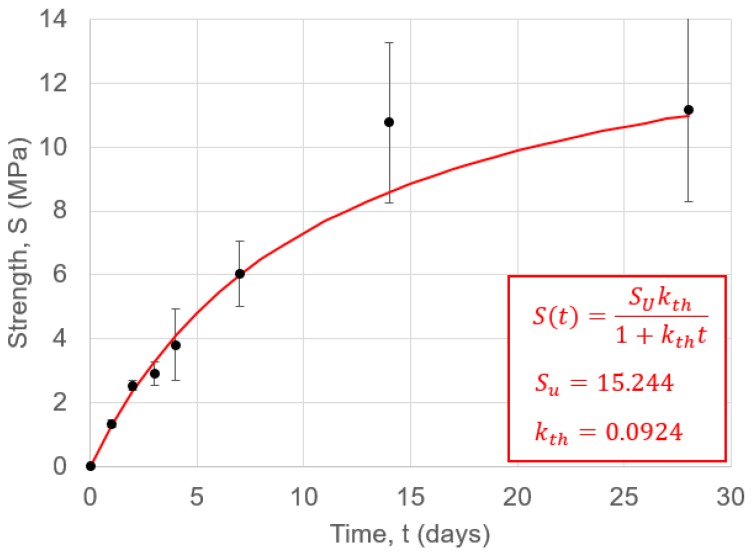
Compressive strength values for geopolymer cubes as a function of time. Error bars show the standard deviation, taken over three cube tests at each time point.

The evolution of the compressive strength of the geoploymer is shown in Figure 7, with a non-linear fit obtained as outlined in [97]. The mean value of compressive strength for 28 days met standard BS EN 1504-3:2005 for a non-structural class R1 repair; however, there was a growing degree of strength variability in samples as they cured. As expected, the evolution of strength was notably slower than for geopolymers cured at elevated temperatures. 

### 4.2. Coating Thickness

**Figure 8:**
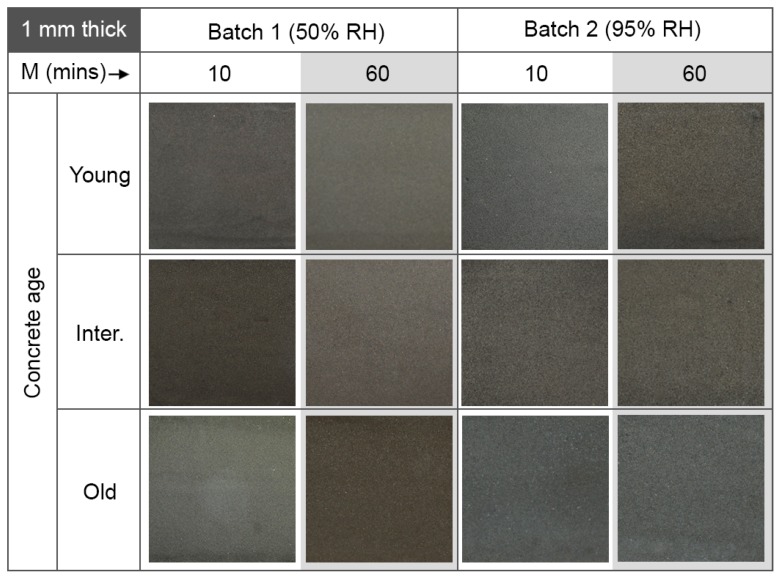
Images of 1 mm thick geopolymer coatings on concrete. Results are shown as a function of geopolymer mixing time, M; relative humidity (RH) during curing; and concrete substrate age. All images shown cover a 40 mm × 40 mm area on the sample. The crack quantification algorithm found negligible cracking in all samples (typically 0.001%).

**Figure 9:**
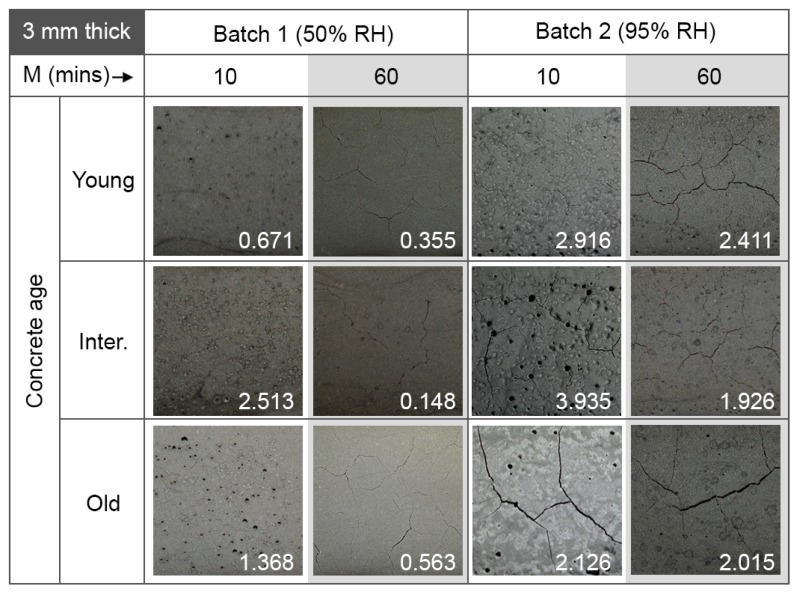
Images of 3 mm thick geopolymer coatings on concrete. Results are shown as a function of geopolymer mixing time, M; relative humidity during curing; and concrete substrate age. All images shown cover a 40 mm × 40 mm area on the sample. The numbers shown inset in each image are the crack percentages generated by the quantification algorithm.

The main finding of this investigation was related to the thickness of the geopolymer coatings applied onto the concrete specimens. These results are shown in Figure 8 and Figure 9 for 1 mm thick and 3 mm thick coatings, respectively. Coatings with a thickness of m = 1 mm (thin coatings), showed no cracks and a good layer integrity, regardless of the age of the concrete substrate, the mixing time or the curing conditions. The algorithm used to detect surface quality gave an average value of 0.001% defects for all samples. On the other hand, geopolymer coatings with a thickness of m = 3 mm (thick coatings) tended to show cracking. The extent of the cracking depends on the mixing time, but was independent of the age of concrete and the curing conditions. The percentage values generated by the crack detection algorithm are shown inset in each image in Figure 9.

This finding was initially surprising: the lower surface/volume ratio of the 3 mm thick coating should allow the geopolymer to retain more water. The result was also at odds with previous work by Zhang et al. [8], who concluded that increasing the thickness of the coatings from 3 mm to 5 mm reduced shrinkage. On the other hand, according to [78], the overall shrinkage of repair material increases with the repair volume, and this same result was found for concrete [98] and cementitious materials in general [99]. The explanation for our results could be that thicker layers show a higher drying shrinkage, since water is well-retained and evaporation takes place more gradually after the geopolymer matrix has slightly hardened (therefore generating stress). The water absorbed in thinner layers, meanwhile, is more likely to evaporate during the first few hours, while the geopolymer is still in a plastic state, and prior to any significant hardening.

### 4.3. Setting and Mixing Time

The thicker coatings in Figure 9 show a relationship between coating quality and mixing time: coatings mixed for 10 min show numerous air bubbles on the surface (black spots). These could be a consequence of unreacted fly ash particles. When mixing times were increased to 1 h, there were fewer air bubbles on the surface and no black spots (fewer unreacted particles). Cracks in thick coatings appeared to be more extensive when the mixing time was longer. It could be that the agglomerates of unreacted fly ash particles acted as ‘micro aggregates’ [89] for the coating, thus enhancing the strength of the coating. However, this hypothesis will require testing in future work.

**Figure 10:**
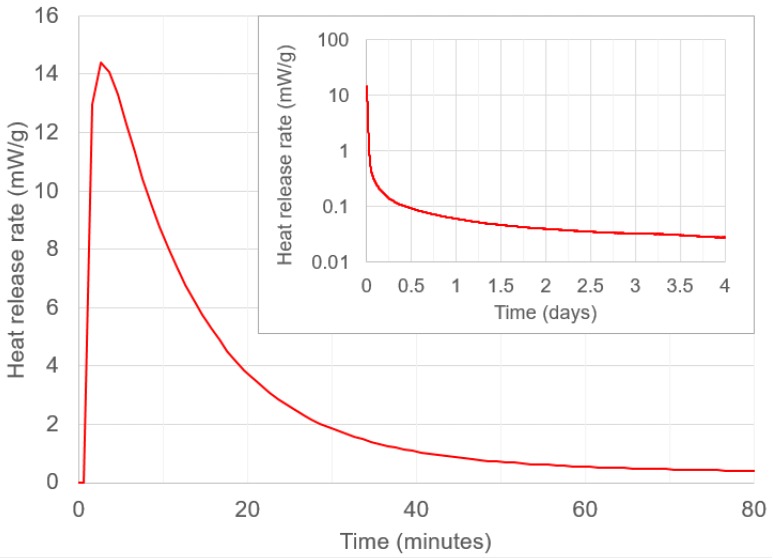
The rate of heat release of the geopolymer over the first 80 min and (inset) over 4 days on a logarithmic scale.

The reason for cracking in these coatings more generally can be explained using Figure 10, which shows the rate of heat release from the geopolymer (obtained using isothermal calorimetry analysis) over 4 days. Rapid heat release occurs within the first hour after mixing. A similar result is seen in [86], for the same liquid to solid ratio. According to [100], heat release can be associated with more shrinkage and cracking in coatings. For an ambient-cured geopolymer, mixing for 1 h is preferable to mixing for 10 mins, as it allows more heat release to happen within the mixing bowl, it allows water to be used in geopolymerisation (rather than being lost to the substrate) and it allows unreacted fly ash particles to dissolve.

**Figure 11:**
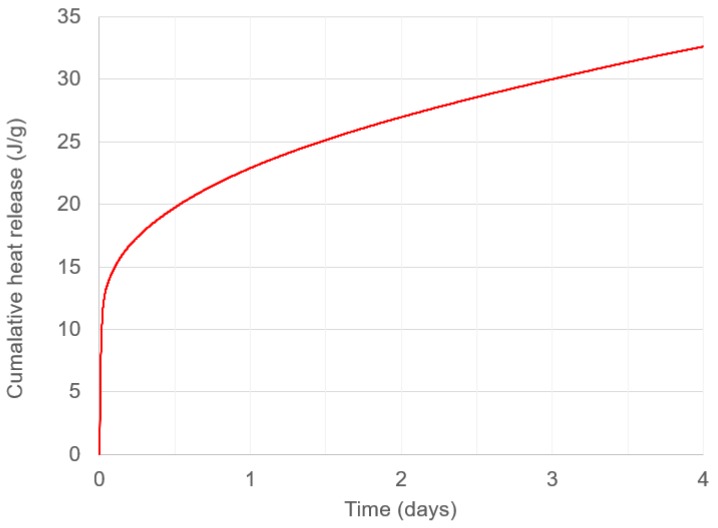
Cumulative heat release of geopolymer binder over 4 days.

Figure 11 shows the cumulative heat release (calculated through cumulative trapezoidal numerical integration of Figure 10). These values grew more gradually than those found in previous work [86], and demonstrated that the reactions occurring in the ambient-cured geopolymer are more gradual. This hypothesis is also supported by the slower strength development shown in Figure 7.

**Figure 12:**
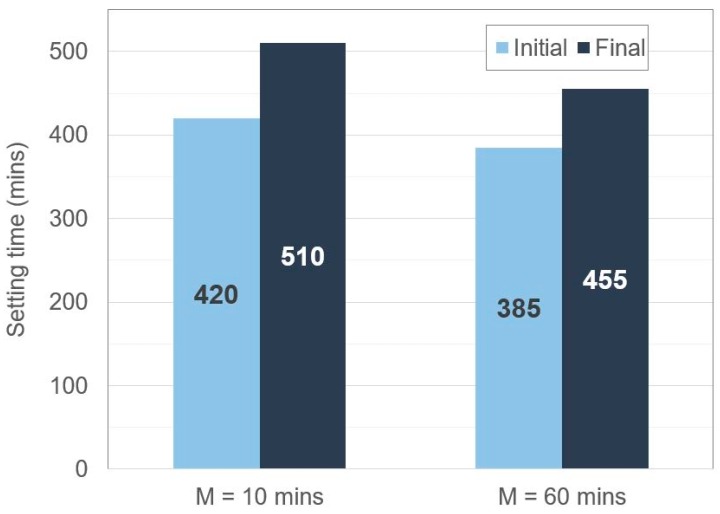
Vicat Needle initial and final setting times of geopolymer samples mixed for M = 10 min and M = 60 min.

Finally, a Vicat Needle test produced the setting times shown in Figure 12. It appears that mixing for longer durations (1 h, as opposed to 10 min) reduced the initial and final setting times, and this also supports the idea that a further extent of geopolymerisation can occur during prolonged mixing. 

### 4.4. Concrete Age

The results in this work showed that the age of the concrete substrate had little to no influence on the integrity of the coating layer: the coating thickness and the mixing time of the geopolymer were far more important factors. However, this does not rule out the effect of concrete age on water absorption from the geopolymer layer in all cases, as the rate-of-hydration and pore-size-change within concrete substrates are strongly dependent on the concrete’s water/cement ratio, the cement particle size and the curing conditions. Nevertheless, this result, together with the independence of the integrity of the coatings on the curing RH levels between 50% and 95%, was encouraging from the standpoint of application: geopolymer coatings can be applied to both new and old concrete assets, and at a wide range of humidity ranges. 

### 4.5. Efflorescence

**Figure 13:**
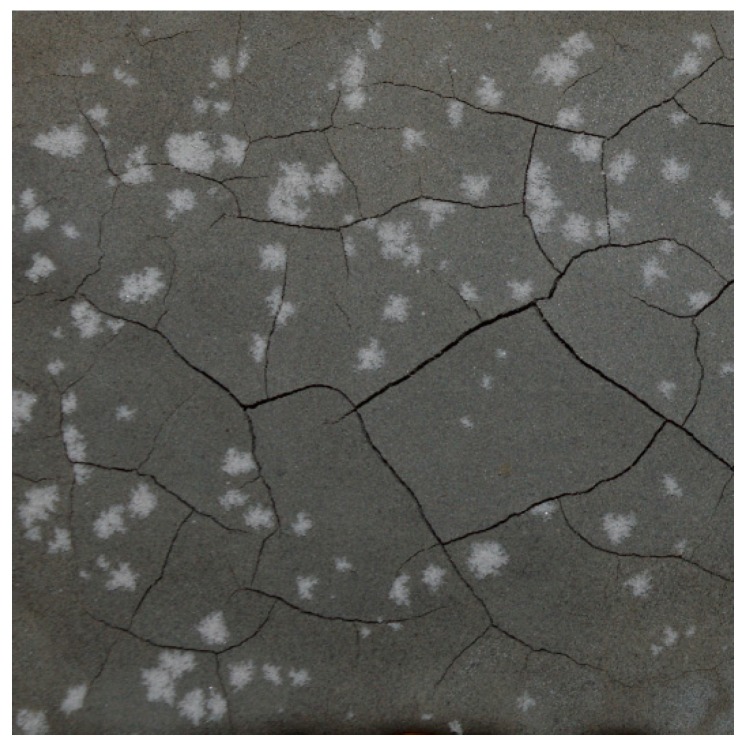
An example of geopolymer coating in batch 2, which demonstrated efflorescence.

The final issue faced in ambient curing of geopolymer coatings on concrete was efflorescence. The samples in batch 2 of Table 3 (high humidity curing) showed, in most cases, evidence of white crystals on the surface 3-8 weeks after application and curing. Efflorescence is a crystallization process, so its extent can change from one sample to the other, but the high humidity increased the propensity for it to occur. Figure 13 shows that efflorescence can be accompanied by the presence of cracking; however, it is likely that efflorescence and cracking are both symptoms of moisture transport within the sample, mainly due to the drying process. 

**Figure 14:**
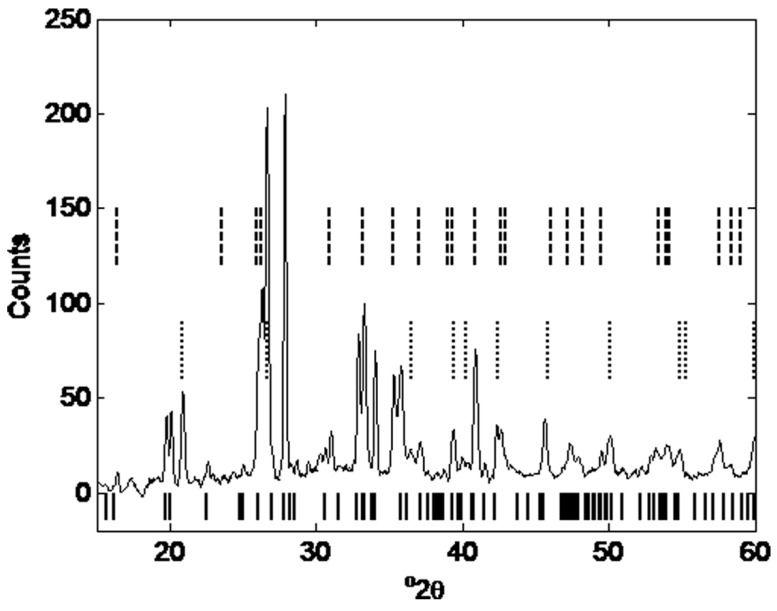
XRD pattern of a geopolymer sample containing efflorescence crystals on the surface: solid black lines below the pattern are gaylussite (Na_2_Ca(CO_3_)_2_·5H_2_O), dotted lines are quartz and dashed lines are mullite.

To further analyze this efflorescence, the samples with efflorescence crystals were analyzed using XRD, as described in Section 3. The results of the XRD analysis of geopolymer layers showing efflorescence are shown in Figure 14. Solid black lines below the pattern are gaylussite (Na_2_Ca(CO_3_)_2_·5H_2_O), dotted lines are quartz and dashed lines are mullite. Background has been removed as the main feature of interest is the crystalline salt. The data was smoothed using the moving average method with a span of 9.

**Figure 15:**
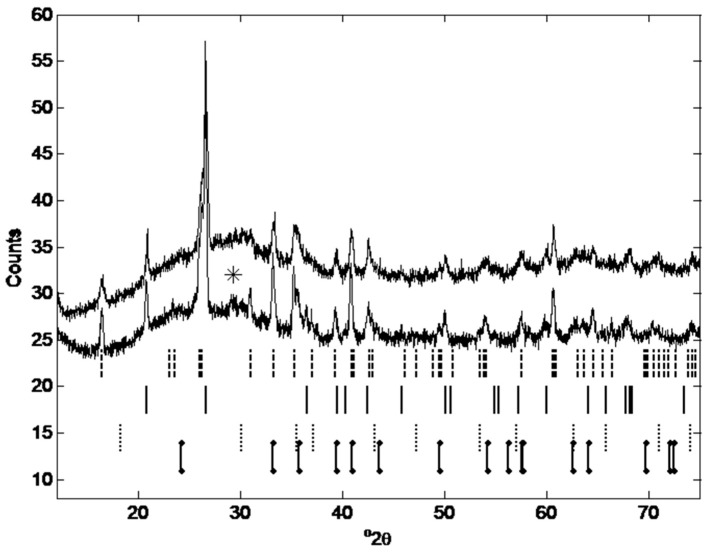
XRD pattern of geopolymer sample of batch 2, Table 2 (upper diffraction pattern), and of the fly ash (lower diffraction pattern). Phase identification starting from the top and moving down: dashed lines are mullite (ICSD collection code 66449), solid lines are quartz (ICSD collection code 100341), dotted lines are magnetite (ICSD collection code 82237), lowest dot-dash lines are hematite (ICSD collection code 82137). The star at 29.4 o2theta is a gypsum reflection (ICSD collection code 15982). The pattern shows an amorphous halo from approximately 20°–35° 2theta, similar to [100].

The results reflected the literature findings discussed in Section 2.2.4. Excess Na_2_O is mobile and at the surface of the coating can react with atmospheric CO_2_ to form Na_2_CO_3_ phases. In our case, the XRD analysis showed that the reaction product was gaylussite—Na_2_Ca(CO_3_)_2_·5H_2_O. This result suggests that Ca may have been dissolved from the concrete substrate or the CaO present in the ash, forming gaylussite at the geopolymer surface. However, the CaO content of the ash is low which is consistent with the lack of visible C-S-H formation in Figure 15 and suggests that Ca more likely came from the concrete substrate. An amorphous halo was present from approximately 25–40 degrees 2theta, consistent with the formation of N-A-S-H, similar to [101,102,103]. 

Figure 15 shows the XRD patterns for a sample of fly ash (lower diffraction pattern), and for a sample of geopolymer obtained from the fly ash (upper diffraction pattern).

The X-ray diffraction (XRD) spectrum of the fly ash and of the geopolymer obtained from the fly ash were acquired using the method detailed in Section 3.2.5.3. Identification of gypsum is tenuous as the peak identified is very small and the other reflections between 12–75° 2theta overlap with more major phases. While the XRD pattern for magnetite is almost identical to maghemite (another iron oxide), the phase present was assumed to be magnetite, as it is more common in UK fly ashes [33]. Using Rietveld refinement (TOPAS v5, Bruker) on samples spiked with an internal standard (10 wt% silicon) the crystalline phases and the amorphous content have been quantified for both the fly ash and the geopolymer as presented in Table 4 (fly ash (a) and geopolymer (a)).

The identification of amorphous content in geopolymers is not trivial as no ‘pure’ X-ray amorphous phase is formed without the presence of crystalline minerals. Recent work by Scarlett and Madsen [104] has shown that the internal standard method can overestimate the amorphous content of samples where there is broad range of MAC (micro-absorption coefficient, cm^2^/g) values in the sample. They [104] worked on purpose made samples of controlled composition and particle size and discovered that the PONKCS method (Partial Or No Known Crystal Structure) produced the most accurate estimation of amorphous content. However Sun and Vollpracht [105] compared the amorphous content of fly ash as determined by PONKCS and the internal standard method and achieved virtually identical results (72.9 wt% and 73 wt% respectively) with the two methods. The mass absorption coefficients of the phases present in the fly ash and geopolymer, as taken from the TOPAS refinement are: Quartz (44.6 cm^2^/g), mullite (32.7 cm^2^/g), gypsum (64.7 cm^2^/g), hematite (214.3 cm^2^/g) and magnetite (221.3 cm^2^/g). The outliers are the iron phases but they are also present in small quantities in the fly ash as shown by the XRF composition given in Table 1. The amorphous content of fly ash is described by Williams and van Riessen [93] as 40–80% and it often found to be over 60% [105,106,107] which fits with our results. For comparison, diffraction patterns were collected again on separate but identical samples. In the second collection, the samples were not rotating, a knife edge collimator was not used, the divergence slit was 0.3° and the Ni β filter was placed in the diffracted beam path. Results from this refinement are labelled as b. 

## 5. Further Discussion and Future Work

This work demonstrated that fly ash geopolymer coatings can be cured at room temperature without any additional grinding steps or additives, provided some conditions can be met on site. While ambient-cured coatings did take longer to cure, they were touch-dry within one day, and were strong enough to form a non-structural class R1 repair within 28 days. Accelerants (such as heat or calcium additives) may be required if the application demands more rapid strength development. The main factors that affected coating integrity were related to the retention of water in the geopolymer during its prolonged curing at ambient temperatures. These were:

- Coating thickness: coatings with thickness < 1 mm showed no cracks, regardless of mixing times and RH levels between 50% and 95%. Coatings with higher thickness showed cracks, the extent of which was dependent on the mixing time;
- Mixing time: for our mix, the optimal mixing time was 1 h, to allow the main extent of geopolymerisation reactions to occur without loss of water or thermal stress, and to ensure that only few fly ash particles were unreacted. Optimizing mixing time can allow geopolymer coatings to overcome water loss induced cracking and to show a homogeneous surface without voids and bubbles.
- Age of concrete: the results showed that coating integrity did not depend on concrete age. This is an important consideration if geopolymer coatings and linings are to be applied to newly cast concrete structures, and not only to old structures which need repair.
- Efflorescence: for low-temperature, but humid curing conditions (above 70% RH), efflorescence was likely, as excess alkaline solution crystallized on the surface of the coating. Efflorescence was less likely for relative humidities at or below 50%.

The challenge of ambient-cured geopolymer coatings is by no means easy to solve, due to the several competing and interconnected reactions and water transport processes: as the geopolymer cures, more N-A-S-H gel is produced, thus resulting in smaller pores, and in some cases in closed pores, and this will change the permeability of the geopolymer and so the water transport inside the geopolymer coating [108].

### 5.1. The Role of Coating Thickness

Coating thickness was found to play a role in coating integrity. The thickness of cementitious coatings on concrete assets can play a direct role on their overall performance for their intended use. Firstly, repairs require high strain capacity to resist strain, and subsequently, cracks [80]. Thicker patches generate lower stresses under drying, therefore minimizing the chance of defects [109]. Patches low in volume are susceptible to high amounts of liquid loss when applied onto dry concrete substrates, which in turn can affect cement hydration and their mechanical properties [65]. Furthermore, thin geopolymer patches have been mentioned to be more crack-prone in marine applications than thicker coatings. However, humidity levels were also mentioned to play a role as coatings of equal thickness showcased different behaviors for different exposure to seawater [8]. Thicker cement patches debonded at higher load values than thinner ones under axial loading [88].

The thickness of patches has also been reported to affect the sensing capabilities of cement-based self-sensing coatings for loading applications. Baeza et al. reported that thinner patches had higher sensing capacity [110], whereas Wang et al. stated that thicker patches gave a higher fractional increase in resistance than thinner patches, which was attributed to greater crack propagation in thicker patches compared to thinner ones [111]. While it is currently rather unclear which of the two provide greater sensing capabilities, it can be assumed that the thickness of self-sensing coatings impacts sensing capabilities.

### 5.2. Thermal Expansion and Bond Strength

Morgan [80] has stated that an ideal repair material should display a similar modulus of elasticity and thermal expansion to the parenting substrate and that it should be compatible with the existing structure (in terms of its adhesion strength, capillary water absorption, dilatation properties and durability). The bond strength and thermal expansion of our ambient-cured mix are factors that still require further investigation. As we are aiming to demonstrate a non-structural repair, for the time being, measurements of elastic modulus are not required (according to BS EN 1504-3:2005). 

### 5.3. Characterisation of the True SiO_2_/Al_2_O_3_ Ratio

The actual compositional ratio SiO_2_/Al_2_O_3_ depends on how much aluminosilicate precursor has reacted and on the final product, since a link has been demonstrated between precursor type characterization and extent of the reaction [112]. In order to have an idea of the SiO_2_/Al_2_O_3_ molar ratio of the final geopolymer, a detailed characterization of the geopolymer sample after curing is required, as described in [112]. It is beyond the scope of this paper, but will be taken into consideration within future work.

## 6. Conclusions 

This paper has outlined the manufacture of ambient-cured geopolymer coatings for concrete, without the use of additives. The most important consideration was the interaction between water-transport processes and the geopolymerisation reaction processes responsible for strength gain. While the geopolymer coatings took longer to cure than a thermally cured specimen, they remain a promising choice for retrofitted concrete repairs, rehabilitation and sensing. Future work should focus on solutions to the issues of efflorescence in humid environments, the influence of concrete substrate water saturation, durability under exposure to a variety of environmental conditions and should define methods for studying the relationship between coating thickness, shrinkage and integrity after prolonged geopolymer mixing.

## Figures and Tables

**Table 1:** Composition and properties of the fly ash used in this work.

Source	West Burton Power Station, Lincolnshire, England (UK)
**Supplier**	CEMEX
**SiO_2_**	52.70
**Al_2_O_3_**	21.70
**Fe_2_O_3_**	7.10
**CaO**	4.10
**Loss on ignition**	4.20
**Na_2_O**	1.10
**K_2_O**	2.50
**SO_3_**	0.90
**MgO**	1.80
**Total phosphate**	0.58
**Free CaO**	0.10
**Median particle size, μm**	10.6

**Table 2:** Surface roughness values for each type of concrete used.

Age of Concrete	Young	Intermediate	Old
**Surface roughness (mm)**	0.097	0.053	0.091

**Table 3:** Curing conditions of geopolymer coatings, divided in two batches.

Batch	Curing Conditions	Temperature °C	Curing Time (days)	Average RH %
**1**	Laboratory bench	20 ± 2	28	50
**2**	Environmental chamber	20 ± 1	28	95

**Table 4:** Composition quantified using TOPAS (v.5 Bruker) for a sample of fly ash and a sample of geopolymer from batch 2 of Table 3. The values for fly ash (a) and geopolymer (a) have been determined from a sample rotating at 30 rpm and using a knife edge collimator. The values for fly ash (b) and geopolymer (b) have been determined from a sample which was not rotating and a knife edge collimator was not used. R_wp_ is the weighted profile R factor.

	Phase Content	*Mullite [%]*	*Quartz [%]*	*Magnetite [%]*	*Hematite [%]*	*Amorphous content [%]*	*Gypsum [%]*	R_wp_
Sample								
*Fly ash (a)*		13.74	2.30	1.24	0.67	80.02	1.97	3.9
*Geopolymer (a)*		9.18	2.09	0.89	0.39	86.15	1.30	3.0
*Fly ash (b)*		15.68	3.48	1.25	1.34	76.65	1.36	2.4
*Geopolymer (b)*		12.13	2.75	1.05	0.80	82.12	1.20	2.2
